# Bioactivities of *Alchemilla alpina* L. Extract on Women’s Reproductive and Metabolic Health: Antioxidant, Enzyme Inhibitory, Receptor Modulatory Properties and Potential Cytotoxic Effects

**DOI:** 10.3390/ijms27073025

**Published:** 2026-03-26

**Authors:** Sanja Krstić, Sofija Bekić, Nemanja Živanović, Andrea Pirković, Jovana Vuković, Rudolf Bauer, Milena Rašeta

**Affiliations:** 1 Institute of Pharmaceutical Sciences, University of Graz, 8010 Graz, Austria; rudolf.bauer@uni-graz.at; 2 Department of Chemistry, Biochemistry and Environmental Protection, Faculty of Sciences, University of Novi Sad, 21000 Novi Sad, Serbia; sofija.bekic@dh.uns.ac.rs (S.B.); nemanja.zivanovic@dh.uns.ac.rs (N.Ž.); milena.raseta@dh.uns.ac.rs (M.R.); 3 Department for Biology of Reproduction, Institute for the Application of Nuclear Energy (INEP), University of Belgrade, 11080 Belgrade, Serbia; andrea.pirkovic@inep.co.rs (A.P.); jovana.vukovic@inep.co.rs (J.V.)

**Keywords:** *Alchemilla alpina*, LC-MS/MS, phenolic compounds, antioxidant activity, α-glucosidase, α-amylase, acetylcholinesterase inhibition, estrogen receptor, aldo-keto reductase, women’s reproductive health

## Abstract

*Alchemilla alpina* L. (Rosaceae), belongs to a genus well recognized in traditional medicine for treating gynecological disorders and hormonal imbalance; however, the specific bioactivity of *A. alpina* itself remains poorly characterized. This study aimed to elucidate the phenolic composition and the biological potential of the methanolic (MeOH) extract of *A. alpina*. LC–MS/MS analysis identified 39 phenolic compounds, with rutin, catechin, kaempferol-3-*O*-glucoside, and caffeic acid being the dominant constituents. The extract exhibited high total phenolic and flavonoid contents, consistent with strong antioxidant capacities. It demonstrated notable α-glucosidase and acetylcholinesterase inhibitory activities, indicating its potential relevance for metabolic and neurodegenerative disorders. The extract effectively reduced AAPH-induced ROS levels in MRC-5 fibroblasts, indicating cytoprotective and antioxidative effects. The cytotoxicity toward cervical cancer cells HeLa and ovarian cancer cells A2780 was moderate and concentration dependent. A yeast-based fluorescent screen revealed a strong and selective binding affinity toward estrogen receptor α (ERα) and selective inhibition of human recombinant AKR1C3 (59.5%), without affecting AKR1C4. Additionally, high COX-1/COX-2 inhibition (>70%) supported its anti-inflammatory potential. Collectively, these findings provide the first integrated evidence of *A. alpina*’s phenolic richness and multifunctional bioactivity, scientifically supporting its potential in managing hormone-dependent and oxidative stress-related disorders.

## 1. Introduction

Species of the genus *Alchemilla* (family Rosaceae), commonly known as “Lady’s Mantle” or “lion’s foot,” are predominantly distributed across Europe and Asia, including northeastern Anatolia (Türkey), northern Iraq, and northwestern Iran. In Europe alone, over 300 species have been described, with major distribution centers located in large mountain ranges such as the Caucasus, the Alps, and the Carpathians, many of which host numerous endemic taxa [1]. These plants have a long history of use in conventional medicine, particularly for promoting wound healing, treating skin disorders, gastrointestinal ailments, and various gynecological conditions [1]. One of the most studied traditional applications is in women’s health, including the alleviation of menopausal discomforts and various gynecological disorders [2,3,4]. The reported benefits include the relief of menstrual cramps, the regulation of the menstrual cycle, support for hormonal balance in the thyroid and reproductive systems, and the improvement of conditions such as cysts, endometriosis, infertility, and blemishes. In Southeast Europe and across the Balkan Peninsula, Lady’s Mantle is a widely recognized medicinal herb traditionally used to address women’s health conditions such as menstrual irregularities, oligomenorrhea, dysmenorrhea, menopausal symptoms, vaginal secretion regulation, polycystic ovary syndrome, and infertility [5,6].

Due to hybrid crossbreeding, genetic variability, and the presence of numerous morphologically similar species, the taxonomic analysis, interpretation, and description of *Alchemilla* taxa are challenging [7]. Although more than 1000 *Alchemilla* species have been described worldwide, only a small fraction has been thoroughly investigated for its chemical composition and biological activities. Of these, approximately 300 species occur in Europe, many of which are endemic to the region [8].

Several *Alchemilla* species are recognized, but *A. vulgaris* L. (*A. xanthochlora* Rothm.) and *A. mollis* (Buser) Rothm. are among the most widespread and studied varieties [9,10]. The aerial and root parts of *Alchemilla* species, which are included in the European Pharmacopeia, have been reported to contain a wide range of phytochemicals with diverse biological activities. Their medicinal use has been documented for treating various disorders [2,4,11,12,13]. However, most of the available information on the biological properties of *Alchemilla* species is derived from ethnobotanical knowledge and traditional applications rather than robust scientific evidence. It is known that *Alchemilla* spp. exhibit strong anti-inflammatory activity, with several studies indicating greater inhibitory potential against cyclooxygenase-2 (COX-2) compared to cyclooxygenase-1 (COX-1) [1,14,15,16]. Due to their high tannin content, these plants are also used for wound healing and to treat various skin conditions such as rashes, burns, eczema, and ulcers. They may protect against UV-induced skin damage, thickening, erythema, and wrinkle formation [12,17,18]. Several reports suggest their potential against various cancers, including prostate, breast, cervical, ovarian, and colorectal adenocarcinoma [13,19]. While *Alchemilla* plants are also used traditionally to treat infections, their antiviral and antimicrobial properties remain poorly studied [20].

Among the many species within the genus, *Alchemilla alpina*, despite its long history in traditional medicine, remains one of the least studied. Scientific data on its phytochemical profile and pharmacological potential, particularly in relation to women’s reproductive health, are scarce. This lack of rigorous pharmacological research represents a significant gap in the literature, making the investigation of *A. alpina* both timely and necessary.

*Alchemilla alpina* L. em. Buser (Rosaceae), commonly known as Alpine Lady’s Mantle, is an arctic–alpine species typically found in alpine meadows at elevations ranging from (750) 1500 m above sea level (a.s.l.) up to 2400 (2600) m a.s.l. It often occurs as a dominant species in the characteristic alpine pasture vegetation, contributing up to 32% of the total grass composition in these ecosystems [21]. The essential oil composition of *A. alpina* has been characterized by Falchero et al. [21], revealing terpenes (36.9%) and alcohols (30.6%) to be the major constituents, corresponding to 47.3 μg/g and 39.2 μg/g fresh weight, respectively. Aldehydes accounted for 15.4% of the oil (19.7 μg/g fresh weight), followed by acids (2.3%; 3.0 μg/g), esters (1.0%; 1.3 μg/g), and unidentified compounds (0.5%; 0.7 μg/g).

In a more recent study, İnci et al. [22] evaluated MeOH extracts from the aerial parts of *A. alpina* for their antioxidant and antimicrobial potential. The extracts demonstrated notable DPPH radical scavenging activity, with inhibition rates ranging from 45.4% to 94.4%. Antimicrobial effects were also observed, with inhibition zones of 8–23 mm against bacterial strains (*Staphylococcus aureus*, *Escherichia coli*, *Klebsiella pneumoniae*, and *Bacillus megaterium*) and fungal species (*Candida albicans* and *C. glabrata*).

To the best of our knowledge, this is the first comprehensive investigation of the phytochemical composition and multifunctional bioactivity of *Alchemilla alpina* L., with the aim of providing a scientific basis for its traditional ethnopharmacological use and exploring its potential relevance to hormone-dependent women’s reproductive health.

The study aimed to characterize the phenolic profile of the MeOH extract using LC–MS/MS, to determine its total phenolic and flavonoid contents, and to evaluate its antioxidant capacity through biochemical (DPPH, ABTS, FRAP, and NO) and cell-based assays. In addition, the potential metabolic relevance of the extract was assessed via α-amylase and α-glucosidase inhibition, while its cholinesterase inhibitory capacity was evaluated using an acetylcholinesterase inhibition assay. Cytotoxic and cytoprotective effects were investigated in non-tumorigenic (MRC-5) and cancer (HeLa and A2780) cell lines, alongside the extract’s capacity to modulate intracellular ROS under oxidative stress. Our primary aim was to establish a preliminary framework for effective concentration ranges and to evaluate baseline cytotoxic and cellular antioxidant responses. HeLa cells, derived from cervical carcinoma, are frequently employed in initial cytotoxicity assessments due to their reproducibility, availability, and relevance to gynecological malignancies, while the A2780 human ovarian cancer cell line was used to evaluate the cytotoxic potential of the extract in ovarian malignances. While A2780 cells express estrogen receptor β (ERβ), the expression of estrogen receptor α (ERα) is generally low or absent, which distinguishes A2780 from some other hormone-sensitive ovarian cancer models. This makes A2780 particularly useful for studying ERβ-specific mechanisms rather than classical ERα-driven pathways. MRC-5 cells are immortalized human lung fibroblasts derived from normal embryonic tissue, and they are widely used as a representative model of non-tumorigenic human cells in toxicology and pharmacology studies. In the current study, MRC-5 cells were used as a comparative non-tumorigenic reference to the cancer cell lines, allowing us to distinguish general cytotoxic effects from tumor-specific responses. Furthermore, the study examined its affinity toward ERα, ERβ, androgen receptor (AR), and glucocorticoid receptor (GR) using a yeast-based fluorescent biosensor, as well as its inhibitory effects on human recombinant aldo-keto reductases 1C3 (AKR1C3) and 1C4 (AKR1C4), and cyclooxygenase enzymes (COX-1 and COX-2). Importantly, this research provides the first report of acetylcholinesterase, α-glucosidase/α-amylase, and the COX inhibitory activities of *A. alpina*, expanding the current understanding of its pharmacological potential. The overall aim was to establish a preliminary framework that supports future mechanistic and *in vivo* research focused on hormone-dependent, metabolic, and oxidative stress-related conditions affecting women’s reproductive health.

## 2. Results and Discussion

### 2.1. Phytochemical Analysis

#### 2.1.1. Total Phenolic (TPC) and Total Flavonoid Contents (TFC) of Tested *A. alpina* Extract

The TPC of the MeOH extract of *A. alpina* was found to be 163.12 ± 1.62 mg GAE/g d.w., indicating a high abundance of polyphenolic compounds (Table 1). The TFC was 40.48 ± 0.49 mg QE/g d.w. (Table 1), suggesting that flavonoids represent a notable fraction of the plant’s secondary metabolites.

The obtained results are much higher compared with those of our previous study on *A. vulgaris* for the same extract type (7.71 ± 0.01 mg GAE/g d.w.) [13], but no comparison with *A. alpina* the form of crude extracts is possible, since there is lack of literature data. Furthermore, this is the first report of TPC and TFC for this plant species, and the obtained values are not only remarkable due to their high content but also show good agreement with the LC–MS/MS-identified phenolic profile presented in Table 2.

#### 2.1.2. Liquid Chromatography–Tandem Mass Spectrometry (LC-MS/MS) Analysis of Tested *A. alpina* Extract

The LC–MS/MS analysis of 43 selected phenolic compounds in *A. alpina* MeOH extract resulted in 39 detected compounds, and 34 of them were fully quantified (Table 2).

The LC–MS/MS analysis of the *A. alpina* MeOH extract revealed a diverse profile of phenolic acids, flavonoids, coumarins, and lignans (Table 2). A total of 39 compounds were detected, of which 34 were quantified, while the others were present at levels below the limit of quantification (LoQ), indicating a rich polyphenolic composition that may contribute to the plant’s pharmacological potential.

Among the hydroxybenzoic acid derivatives, gallic acid (96.99 ± 7.14 µg/g d.w.) was the most abundant, followed by *p*-hydroxybenzoic acid (67.12 ± 0.76 µg/g d.w.) and protocatechuic acid (54.93 ± 0.46 µg/g d.w.). These compounds are well known for their antioxidant and anti-inflammatory activities [23], with gallic acid in particular linked to anticancer effects through apoptosis induction and cell cycle arrest [24]. Hydroxycinnamic acid derivatives were dominated by caffeic acid (181.28 ± 4.37 µg/g d.w.) and chlorogenic acid (163.99 ± 53.81 µg/g d.w.), while ferulic acid (58.09 ± 0.98 µg/g d.w.) and *p*-coumaric acid (55.60 ± 1.14 µg/g d.w.) were also notable. These phenolic acids are associated with antioxidant, anti-inflammatory, and enzyme-inhibitory properties. In addition, caffeic acid has been reported to modulate estrogen receptor activity [25], which is particularly relevant to women’s reproductive health [26]. Flavonoids represented the largest and most diverse class of polyphenols. Rutin was the predominant compound (527.83 ± 8.31 µg/g d.w.), followed by kaempferol-3-*O*-glucoside (318.30 ± 11.26 µg/g d.w.), catechin (347.16 ± 12.15 µg/g d.w.), and quercetin-3-*O*-glucoside (127.96 ± 0.62 µg/g d.w.). These flavonols are recognized for their strong radical scavenging capacity, vascular-protective effects, and chemopreventive potential. Among flavones, luteolin-7-*O*-glucoside (123.84 ± 3.02 µg/g d.w.) and luteolin (44.79 ± 1.23 µg/g d.w.) were the most abundant, both compounds associated with anti-inflammatory, anticancer, and estrogen-modulating properties. Apigenin and its glycosides, though present at lower concentrations, are also reported to exert anti-proliferative and anti-metastatic effects in hormone-related cancers [27].

Coumarins were detected at relatively low levels, with esculetin (10.85 ± 0.18 µg/g d.w.) as the major representative. Esculetin is known for its antioxidant, anti-inflammatory, and anti-melanogenic effects. Lignans were nearly absent, with only trace amounts of secoisolariciresinol detected.

The predominance of rutin, catechin, kaempferol derivatives, caffeic acid, and chlorogenic acid suggests a strong antioxidant, anti-inflammatory and cytoprotective potential of *A. alpina* [24,25,26,27]. Several of these compounds—particularly flavonols, luteolin, and hydroxycinnamic acids—are reported to interact with hormone-related pathways, supporting the traditional use of *A. alpina* in women’s reproductive health [25]. The high rutin content is especially notable, as rutin has been linked to vascular health and the modulation of estrogenic activity, both of which are relevant in gynecological disorders [28].

Overall, the quantitative profile confirms that *A. alpina* is a rich source of bioactive polyphenols. These findings align with the hypothesis that its traditional medicinal uses may be attributed, at least in part, to its complex and potent phenolic composition. The dominance of flavonols and hydroxycinnamic acids provides a biochemical basis for the observed antioxidant and potential anticancer activities, warranting further functional assays and *in vivo* validation.

In comparison with our previous study on *A. vulgaris* [13], distinct phenolic profiles were observed depending on the extraction solvent. While the MeOH extract of *A. alpina* showed relatively high amounts of quantified polyphenolics, *A. vulgaris* ethyl acetate extract was the richest source of phenolics overall, with catechin (8144.98 ± 0.01 μg/g d.w.) being the most abundant compound, whereas luteolin-7-*O*-β-glucoside (903.94 ± 0.03 μg/g d.w.) predominated in the MeOH extract [13].

To the best of our knowledge, there are no previous data on the phenolic profile of *A. alpina*. In contrast, an overview of phenolic compounds across the *Alchemilla* genus identified 94 compounds, including quinic acid [1]. The comparison of our LC–MS/MS findings with this review revealed that sixteen compounds overlapped between *A. alpina* and other *Alchemilla* species, including kaempferol, quercetin, rutin, hyperoside, isoquercitrin, quercitrin, isorhamnetin, luteolin, apigenin, vitexin, chrysoeriol, catechin, epicatechin, naringenin, genistein, and daidzein. This overlap demonstrates that *A. alpina* shares a substantial portion of its phenolic profile with congeners, particularly flavonols (quercetin and its glycosides, kaempferol, and isorhamnetin), flavones (luteolin, apigenin, vitexin, and chrysoeriol), and flavan-3-ols (catechin and epicatechin). Such compounds likely contribute to the strong antioxidant and bioactive properties characteristic of the genus.

### 2.2. Bioactivities of Tested Extract

#### 2.2.1. *In Vitro* Antioxidant Activity of Tested *A. alpina* Extract

The MeOH extract of *A. alpina* demonstrated strong *in vitro* antioxidant potential in all the tested assays (Table 1). The results presented in Table 1 show that the differences between the MeOH extract of *A. alpina* and propyl gallate are statistically significant (*p* < 0.05). The DPPH radical scavenging activity showed an IC_50_ value of 0.01188 ± 0.001 mg/mL, indicating high radical neutralization efficiency. Similarly, the ABTS radical scavenging activity reached IC_50_ of 0.032 ± 0.001 mg/mL, while the ferric reducing antioxidant power (FRAP) was measured at 158.46 ± 13.25 mg AAE/g d.w. Nitric oxide (NO) scavenging activity was also evident, with an IC_50_ value of 1.15 ± 0.24 mg/mL, supporting the overall antioxidant efficacy of the extract. These findings are consistent with the LC–MS/MS profile (Table 2), which revealed high levels of rutin (527.83 ± 8.31 µg/g d.w.), catechin (347.16 ± 12.15 µg/g d.w.), kaempferol-3-*O*-glucoside (318.30 ± 11.26 µg/g d.w.), and caffeic acid (181.28 ± 4.37 µg/g d.w.), compounds widely recognized for their potent radical scavenging and reducing capacities. The abundance of hydroxycinnamic acids (chlorogenic and ferulic acid) and flavones such as luteolin and luteolin-7-*O*-glucoside further supports the strong antioxidant response of the tested *Alchemilla* sp. These phenolic acids and flavones are recognized for their ability to inhibit oxidative stress through various mechanisms, including metal chelation and the suppression of pro-oxidant enzymes. To the best of our knowledge, this study provides the first documentation of the antioxidant profile of MeOH extract from *A. alpina*, with the exception of the DPPH assay. In this regard, only one previous study examined the DPPH scavenging ability of Turkish *A. alpina* samples, reporting inhibition values ranging from 45.4% to 95.4% at concentrations of 1.25–10 mg/mL. By contrast, our extract achieved 94.76% inhibition at a much lower concentration (0.1 mg/mL), demonstrating stronger activity than the results reported by İnci et al. [22]. This pronounced antioxidant effect can be attributed to the high levels of rutin, catechin, caffeic acid, and chlorogenic acid detected in the extract, which are known for their potent radical scavenging and reducing properties.

#### 2.2.2. α-Amylase and α-Glucosidase Inhibitory Activity of Tested *A. alpina* Extract

The MeOH extract of *A. alpina* exhibited inhibitory effects against both α-amylase and α-glucosidase enzymes under *in vitro* conditions (Table 3). These assays provide preliminary insight into the interaction of extract constituents with key carbohydrate-hydrolyzing enzymes but do not allow for conclusions to be drawn regarding the pharmacological or hypoglycemic efficacy *in vivo*.

At the tested concentrations, α-amylase inhibition by the MeOH extract was low (8.30 ± 0.46%) compared to the reference inhibitor acarbose (72.41 ± 6.12%). In contrast, the extract exhibited moderate α-glucosidase inhibition (48.34 ± 2.90%), exceeding that of acarbose (28.06 ± 2.66%) under the applied assay conditions. Notably, the extract was evaluated at a six-fold lower working concentration than acarbose, which may partially account for the observed differences in inhibitory efficacy. These results indicate that the extract interacts more effectively with α-glucosidase than with α-amylase *in vitro*; however, such enzyme inhibitory activity alone cannot be directly translated into antidiabetic or hypoglycemic pharmacological effects without validation in cellular or *in vivo* models.

The observed enzyme inhibition can be attributed to the phenolic composition of the extract, particularly caffeic acid, chlorogenic acid, rutin, and quercetin derivatives, which are known to suppress carbohydrate-hydrolyzing enzymes and modulate glucose absorption [29,30]. Among these, rutin (527.83 ± 8.31 µg/g d.w.) and catechin (347.16 ± 12.15 µg/g d.w.), identified as the most abundant flavonoids in the extract (Table 1), likely play a central role. Rutin decreases carbohydrate absorption by inhibiting α-glucosidase, enhances glucose uptake and insulin secretion, protects pancreatic islets, and reduces oxidative stress and inflammation [31,32,33]. Catechin improves insulin sensitivity by activating PI3K/AKT and AMPK pathways, it promotes GLUT4 translocation and glycogen synthesis, and, together with rutin, it synergistically regulates glucose metabolism while preventing diabetes-related complications [33].

Overall, the enzyme inhibition data suggest that *A. alpina* extract contains bioactive constituents capable of interacting with α-glucosidase *in vitro*. These findings should be interpreted as preliminary *in vitro* indicators and cannot be directly extrapolated to *in vivo* pharmacological efficacy. Further studies employing relevant cell-based systems (e.g., adipocytes or hepatocytes) and *in vivo* models are required to clarify the physiological relevance and potential metabolic effects of these findings.

#### 2.2.3. Anti-Acetylcholinesterase Activity of Tested *A. alpina* Extract

The MeOH extract of *A. alpina* exhibited pronounced anti-acetylcholinesterase activity, with an inhibition value of 94.94 ± 0.65%, comparable to that of the reference inhibitor eserine (95.51 ± 1.12%) (Table 3). Although eserine was tested at a substantially lower concentration (0.069 ng/mL) than the extract (0.5 mg/mL), the extract demonstrated a strong enzyme inhibitory capacity under the applied *in vitro* conditions. This result indicates a high affinity of extract constituents toward acetylcholinesterase; however, it should be emphasized that enzymatic inhibition alone does not allow for conclusions to be drawn regarding neuroprotective efficacy, which requires confirmation in cellular or *in vivo* models.

This activity can be attributed to the phenolic constituents identified in the extract, particularly quercetin, luteolin, rutin, and catechin, which are well-documented acetylcholinesterase inhibitors [34,35]. These compounds act through both direct enzyme inhibition and antioxidant mechanisms that protect neuronal cells from oxidative stress and degeneration. Although present in lower amounts, apigenin and its glycosides may also contribute to the observed bioactivity, given their reported antioxidant and anti-inflammatory properties. Previous studies demonstrated that flavonoids such as quercetin and luteolin interact effectively with the active site of acetylcholinesterase, accounting for their strong inhibitory activity [34]. Furthermore, rutin, catechin, and apigenin derivatives have been reported to reduce oxidative stress and modulate key cellular signaling pathways, including PI3K/AKT and MAPK, highlighting their broad biological activity and pharmacological potential [36]. In addition, recent findings indicate that small-molecule natural products such as isocoumarin analogs can modulate intracellular signaling pathways, including TrkB-related and MAPK signaling, thereby influencing various cellular responses [37]. While several of these phenolic compounds have been reported elsewhere to exert biological effects through antioxidant activity and the modulation of intracellular signaling pathways (e.g., PI3K/AKT and MAPK) [36], such mechanisms were not investigated in the present study. Therefore, the current findings should be interpreted strictly as evidence of *in vitro* acetylcholinesterase inhibition.

To the best of our knowledge, this is the first report describing the anti-acetylcholinesterase activity of a MeOH extract from *A. alpina*. Further studies employing appropriate neuronal cell models and *in vivo* systems are required to evaluate the biological relevance of these observations in neuronal systems.

#### 2.2.4. Evaluation of Cytotoxic Potential of *A. alpina* Extract in MRC-5, HeLa and A2780 Cells

**Figure 1:**
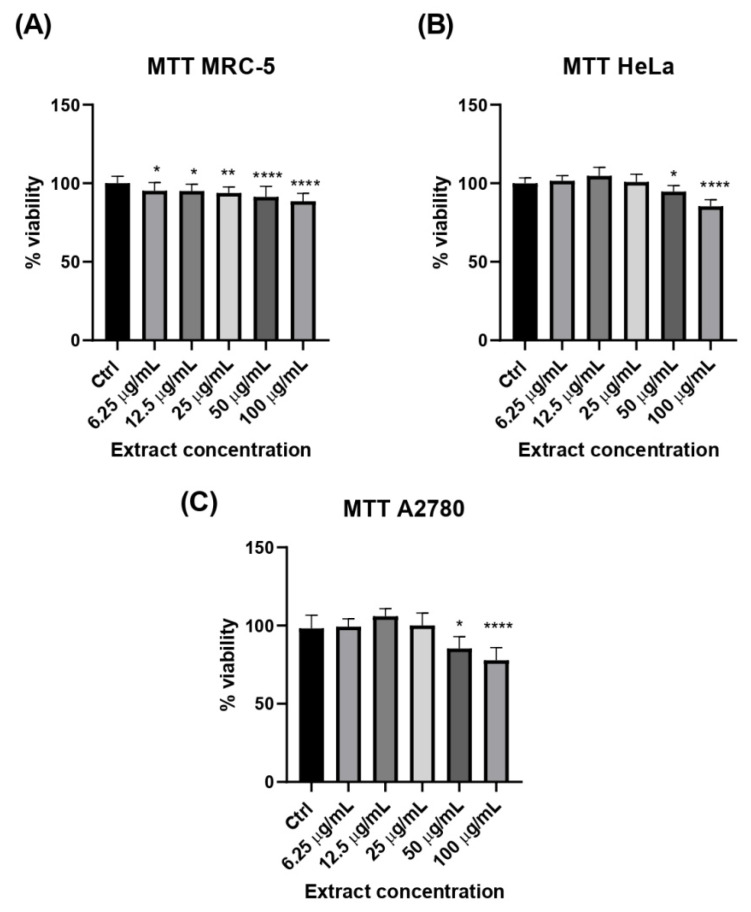
Cytotoxicity of MeOH extract of *Alchemilla alpina* in a range of concentrations (6.25, 12.5, 25, 50 and 100 μg/mL) determined by the MTT assay in the (**A**) non-tumorigenic human fetal lung fibroblasts (MRC-5), (**B**) human cervical carcinoma cell line (HeLa) and (**C**) ovarian cancer cell line (A2780). Data represent the mean ± standard deviation (SD) (* *p* < 0.05, ** *p* < 0.01, and **** *p* < 0.0001 vs. Ctrl) by a one-way analysis of variance (ANOVA) with Tukey’s multiple comparison post hoc test, n = 9.

After 24 h of exposure to the extract across a range of concentrations, a clear concentration-dependent effect on cell viability was observed in both non-tumorigenic MRC-5 and cancer cell lines HeLa and A2780 (Figure 1).

MRC-5 cells showed a consistent decline in viability with increasing extract concentrations, although the decrease was relatively modest compared to the untreated controls (12% reduction in viability at the highest concentration of 100 μg/mL) (Figure 1A). In contrast, both HeLa and A2780 cells exhibited no significant change in viability at concentrations up to 25 μg/mL. However, a consistent reduction in viability was evident at concentrations of 50 μg/mL and above, reaching 15% reduction in viability at the highest concentration of 100 μg/mL in HeLa and 23% viability reduction in A2780 cells (Figure 1B,C). These results indicate a similar threshold concentration at which the extract begins to exert cytotoxic effects, supporting a concentration-dependent mechanism of action. In previous research on *A. vulgaris* extracts, on hormone-dependent and hormone-independent cancer cell lines (human breast MCF7, ovarian A2780, cervical HeLa and prostate cancer PC-3 cell lines), the results revealed that the ethyl acetate (EA) extract showed the strongest tumor growth suppression across all tested lines [13]. While MeOH and ethanol extracts were moderately effective—particularly against MCF-7 and A2780 cells—they did not significantly affect non-tumorigenic cells, indicating selective cytotoxicity toward malignant phenotypes. Notably, even aggressive, hormone-independent cell lines such as HeLa and PC-3 responded to the extracts, suggesting potential therapeutic relevance. The detected IC_50_ in HeLa cells was 80.6 ± 6.3 μg/mL while in A2780 cells’ IC_50_ was 27.9 ± 1.9 μg/mL for *A. vulgaris* MeOH extract. Our current results on the cytotoxicity of *A. alpina* MeOH extract in HeLa and A2780 cells showed IC_50_ > 100 μg/mL for both cell lines. However, it should be noted that the treatments in our study were of a 24 h duration, whereas in the previous study with *A. vulgaris* MeOH extract the incubation period was 72 h. This difference in exposure time may also influence cytotoxicity and could explain the differences in IC_50_ values. Another study that explored the differences in the cytotoxicity of *A. vulgaris* herb extract in non-tumorigenic CCD 841 CoN epithelial cells and colorectal cancer cells (HT-29), following the biotransformation of the extract’s constituents, showed that their cytotoxicity to cancer cells was within a range of concentrations from 50 to 250 μg/mL, where the toxicity to non-tumorigenic cells was not observed. Also, the treatment with the extracts that were not biotransformed showed no cytotoxic effect for both non-tumorigenic and cancer cells [38]. Another study on *A. holotricha* extract showed cytotoxicity against prostate cancer cells PC-3 in a range of concentrations from 125 to 1000 µg/mL, where the cytotoxic activity of the MeOH extract was more profound than that of hexane, dichloromethane, ethyl acetate, and water extracts [39]. Our current results showed that both non-tumorigenic MRC-5 and cancer cells HeLa and A2780 exhibit non-selective reduction in cell viability following the treatment with *A. alpina* MeOH extract, and that concentrations above 50 μg/mL was cytotoxic in all tested cell lines. Collectively, these data indicate that the choice of solvent significantly influences the biological activity of *Alchemilla* sp. extracts, while potential effects of gut microbiota biotransformation should be considered only in light of the previous literature, as this aspect was not investigated in the present study.

The choice of cell lines in this study presents certain limitations with respect to modeling women’s reproductive health. HeLa cells, although derived from cervical carcinoma, are HPV18 transformed and exhibit very low estrogen receptor expression, which restricts their relevance for hormone-mediated pathways. A2780 ovarian carcinoma cells, on the other hand, predominantly express ERβ but lack significant ERα, making them useful for studying ERβ-specific mechanisms but less representative of classical estrogen-responsive cancers. Consequently, while these models provide valuable insights into cytotoxicity and receptor modulation, they do not fully capture the complexity of hormone-sensitive and hormone-insensitive pathways relevant to women’s reproductive health. Thus, the potential of *A. alpina* extract in hormone-dependent reproductive cancers is yet to be fully explored, and future studies incorporating hormone-sensitive and hormone-insensitive models will be necessary to establish this link more clearly.

#### 2.2.5. Preventive Effects of *A. alpina* Extract Against ROS Generation in MRC-5 Cells

**Figure 2:**
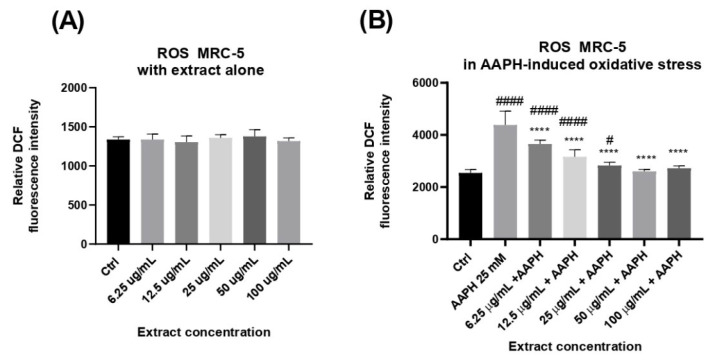
The effect of increasing concentrations of *Alchemilla alpina* extract (at concentrations of 6.25, 12.5, 25, 50 and 100 μg/mL) on intracellular ROS levels in MRC-5 cells, measured by the DCF fluorescence, under two conditions: (**A**) the extract alone without oxidative stress induction and (**B**) in the presence of AAPH-induced oxidative stress. Data represent the mean ± standard deviation (SD) (**** *p* < 0.0001 vs. AAPH; (# *p* < 0.05; #### *p* < 0.0001 vs. Ctrl) by a one-way analysis of variance (ANOVA) with Tukey’s multiple comparison post hoc test; n = 9.

To evaluate the cytoprotective effects of *A. alpina* extract against AAPH-induced oxidative stress in non-tumorigenic MRC-5 cells, intracellular reactive oxygen species (ROS) levels were measured following treatment with a range of extract concentrations. It could be observed that the 24 h treatment with the extract alone did not influence the ROS production in cells per se (Figure 2A). Next, the effects of the extract under conditions of AAPH-induced oxidative stress were evaluated. As illustrated in Figure 2B, ROS levels in the AAPH-treated group were significantly elevated compared to the untreated control, indicating increased oxidative stress. However, co-treatment with varying concentrations of *A. alpina* extract led to a marked, concentration-dependent reduction in ROS levels relative to the AAPH group. All tested concentrations demonstrated antioxidant activity by lowering intracellular ROS production, with the highest concentrations (50 and 100 μg/mL) restoring the ROS levels to those observed in the untreated control group. Extracts from *Alchemilla* sp. have previously shown antioxidant properties in various types of cells. *Alchemilla mollis* (Buser) Rothm. (Rosaceae) aerial parts extracts showed DPPH^·^ and ABTS^+^ radical scavenging as well as the inhibition of the NO production in RAW 264.7 cells at concentration 100 μg/mL [40]. In another study, the EtOH extracts of *A. mollis* exhibited strong antioxidant activity, efficiently neutralizing ABTS and DPPH radicals and inhibiting intracellular ROS production in UVB-irradiated non-tumorigenic human fibroblasts at concentrations of 10 and 100 μg/mL [17]. A protective effect was attributed to the presence of phenolic compounds and gallic acid within the plant extract, which act as antioxidants, scavenging free radicals and activating cellular defense pathways like Nrf2/ARE signaling. This is in accordance with our findings of decreased ROS production following incubation with *A. alpina* MeOH extract in AAPH-exposed MRC-5 fibroblasts.

#### 2.2.6. Relative Binding Affinities of *A. alpina* MeOH Extract for the Ligand Binding Domains of Estrogen Receptor α, Estrogen Receptor β, Androgen Receptor, and Glucocorticoid Receptor

**Figure 3:**
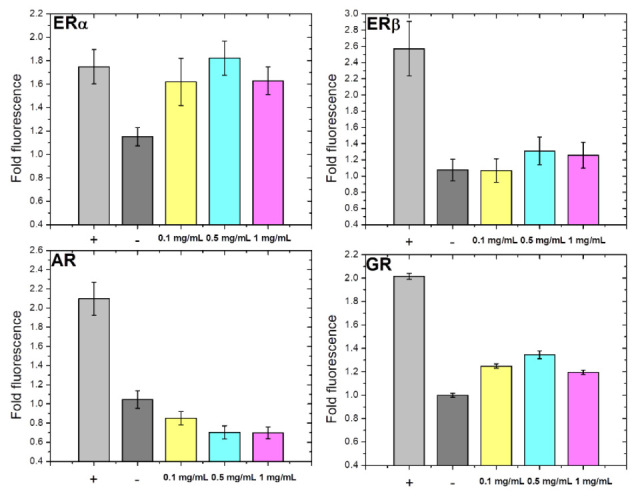
Relative binding affinities of *A. alpina* MeOH extract at three tested concentrations (0.1, 0.5, and 1 mg/mL) for ligand-binding domains of estrogen receptor α (ERα), estrogen receptor β (ERβ), androgen receptor (AR) and glucocorticoid receptor (GR) measured by fluorescent screen in yeast. Relative binding affinities were expressed as fold fluorescence change between ligand-treated cells and control cells treated with DMSO (ER and AR assays) or estradiol (GR assay), normalized to one. Positive/negative controls: estrone/androstenedione (ERα and ERβ), androstenedione/estrone (AR) and prednisolone/estradiol (GR). Control ligands were used at 10 µM in ERα/ERβ assays, and at 100 µM in the AR/GR assays.

Interest in plant-derived natural compounds with anti-estrogen properties has been growing due to their potential in the treatment of endocrine-related disorders [41]. In this study, the relative binding affinities of *A. alpina* extract for the ligand-binding domains (LBDs) of estrogen receptor α (ERα), estrogen receptor β (ERβ), androgen receptor (AR) and glucocorticoid receptor (GR) were evaluated using a yeast-based fluorescent biosensor, as previously described [24,42,43]. This assay has been successfully applied for screening libraries of steroid derivatives [44,45], bile acids [46], as well as plant [43] and fungal extracts [24]. The principle of the assay is based on the expression of LBD of steroid receptor fused to yellow fluorescent protein (YFP) in yeast. Upon the addition of the ligand, receptor dimerization occurs, enabling fluorescence resonance energy transfer (FRET) between YFP molecules, which is detected as an increase in fluorescence intensity. To the best of our knowledge, this is the first report on the steroid receptor binding affinities of *A. alpina* MeOH extract. In this study, the results are presented for the three tested concentrations of the extract (0.1, 0.5, and 1 mg/mL), allowing for the evaluation of its concentration-dependent effects in the yeast biosensor. At a lower concentration (0.1 mg/mL) no detectable binding was observed, while at a higher concentration (1 mg/mL) the signal decreased, likely due to potential cytotoxic or stress-related effects in the yeast biosensor system. The 0.5 mg/mL concentration provided the most reliable and reproducible response, and therefore the subsequent discussion focuses primarily on this concentration, as it most accurately reflects the extract’s receptor-binding potential. As shown in Figure 3, at the 0.5 mg/mL concentration the extract exhibited strong and selective binding affinity to ERα-LBD, with similar fold fluorescence enhancement (1.82) as the natural ligand, estrone (1.75), suggesting that its bioactive molecules may modulate ERα-mediated signaling pathways.

These findings provide molecular evidence that extracts from *Alchemilla* species interact with estrogen receptors, which may underlie their traditional use in managing female reproductive health conditions [47]. The evaluated estrogenic potential of *A. alpina* extract in this study is consistent with reported high content of polyphenolic compounds with estrogenic properties in *Alchemilla* species [1,15]. The observed estrogenic activity is likely the result of the combined action of multiple polyphenolic constituents present in the extract rather than the effect of a single compound. Genistein, a well-characterized isoflavone with affinity for both ERα and ERβ was detected at low levels in the extract (1.89 ± 0.09 µg/g d.w.; Table 2) and serves as a representative marker of the estrogen-related phytochemical profile of *A. alpina*. In our previous work [43], we demonstrated that genistein and its related isoflavones bind to ERβ-LBD with affinities comparable to estradiol, providing mechanistic context for the estrogen-responsive behavior of polyphenol-rich extracts, even when individual constituents are present at low concentrations. These findings align with other studies reporting that genistein behaves as a weak estradiol agonist capable of modulating transcriptional activity through both ER isoforms in human osteoblast models [48]. The extract exhibited weak binding to GR-LBD, lower than the affinity of the standard drug prednisolone, and no interaction with ERβ- and AR-LBD, indicating the safety profile by minimizing the off-target hormonal effects. Selective binding to ERα is important because this receptor regulates reproductive health, bone metabolism and cardiovascular function, although further studies are needed to determine whether compounds from the extract act as agonists or antagonists. Furthermore, ERα is commonly overexpressed in hormone-dependent breast, endometrial and ovarian cancers, malignancies that significantly affect women’s reproductive health, and expression of this isoform is associated with increased tumor growth. Conversely, ERβ plays a role in suppressing proliferation and reducing the effects of ERα signaling [49]. Overall, the results from this preliminary *in vitro* screening indicate that *A. alpina* extract may represent a promising candidate for modulating ERα pathways in reproductive tissues; however, further studies, including *in vivo* investigations, are required to fully elucidate its biological effects and therapeutic potential.

#### 2.2.7. *In Vitro* Inhibition of Human Recombinant AKR1C3 and AKR1C4 Activity by *A. alpina* MeOH Extract

**Figure 4:**
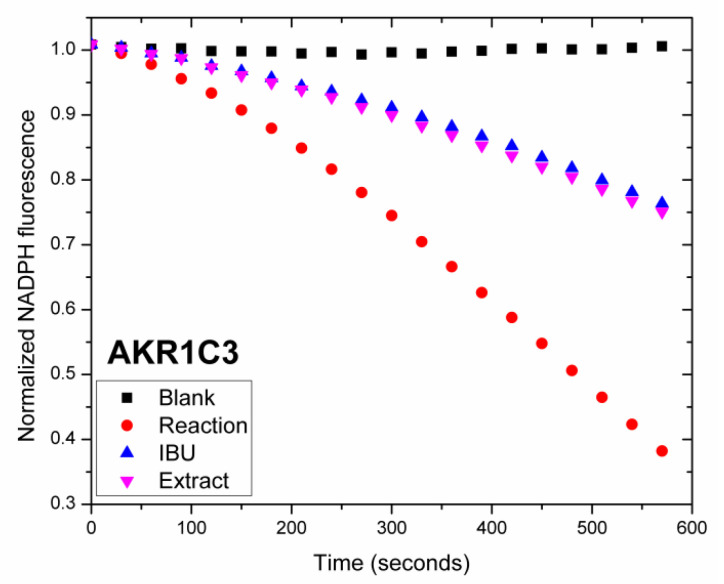
*In vitro* AKR1C3 inhibitory potential of *A. alpina* MeOH extract evaluated by NADPH consumption assay. Change in normalized NADPH fluorescence over time during reduction of 9,10-phenanthrenequinone by human recombinant AKR1C3 in the absence (Reaction) or presence of either the extract (Extract) or ibuprofen (IBU). Fluorescence values were normalized to the initial value and fitted by linear regression yielding curves starting from normalized value one. Blank represents a control probe in the absence of enzyme.

**Figure 5:**
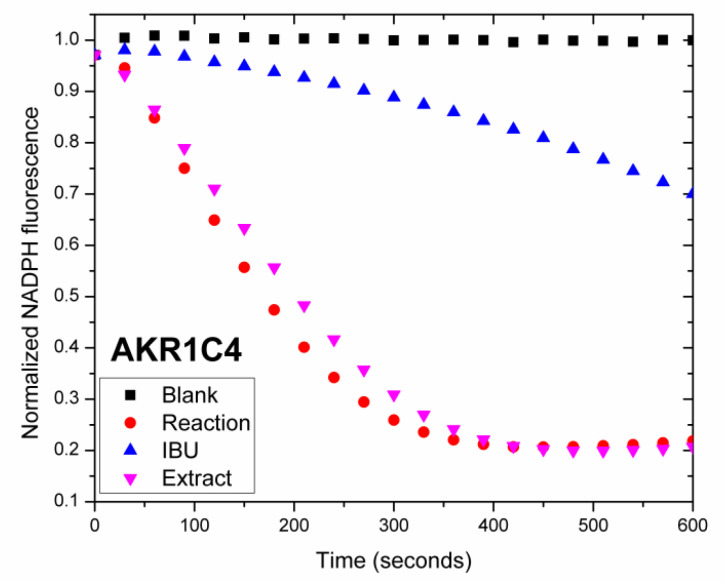
*In vitro* AKR1C4 inhibitory potential of *A. alpina* MeOH extract evaluated by NADPH consumption assay. Change in normalized NADPH fluorescence over time during reduction of 9,10-phenanthrenequinone by human recombinant AKR1C4 in the absence (Reaction) or presence of either the extract (Extract) or ibuprofen (IBU). Fluorescence values were normalized to the initial value and fitted by linear regression yielding curves starting from normalized value 1. Blank represents a control probe in the absence of enzyme.

Human aldo-keto reductases (AKR1C1-AKR1C4) act as 3-, 17- and 20-ketosteroid reductases, modulating the activity of androgens, estrogens and progesterone, as well as the occupancy of their corresponding steroid receptors. Among these, AKR1C3, also referred to as type 5 17β-hydroxysteroid dehydrogenase (17β-HSD5) and prostaglandin F2α synthase, catalyzes the conversion of Δ4-androstene-3,17-dione to testosterone, 5α-androstane-3,17-dione to 5α-dihydrotestosterone (DHT), and estrone to 17β-estradiol, increasing the local levels of potent androgens while also promoting the inactivation of DHT into less active metabolites. AKR1C4, although primarily expressed in the liver, efficiently reduces DHT and is closely related to AKR1C3. Other family members, such as AKR1C2, also modulate local steroid hormone levels [50,51], but in this study we focus on the inhibition of the AKR1C3 and AKR1C4 isoforms. The selective inhibition of AKR1C3 may represent a potential therapeutic strategy in estrogen-driven breast cancer, endometriosis, and conditions requiring enhanced progesterone action such as pregnancy maintenance [50]. Moreover, the oxidoreductase activity of AKR1C3 contributes to resistance to certain chemotherapeutics, such as anthracyclines [51]. In this study the potential of *A. alpina* MeOH extract to inhibit human recombinant AKR1C3 and AKR1C4 was measured by monitoring NADPH consumption using fluorescence spectroscopy, as previously described [25,44,45]. To our knowledge, this is the first study to investigate the inhibitory effects of *A*. *alpina* extract on the activity of aldo-keto reductases. To evaluate the inhibition of *A. alpina* extract, we measured its effect on the reduction of 9,10-phenanthrenequinone catalyzed by recombinant AKR1C3 and AKR1C4, and the results are shown in Figure 4 and Figure 5.

The extract showed strong inhibition of AKR1C3 activity (59.5%), comparable to that of ibuprofen (61.97%), while exhibiting no inhibitory effect on liver-specific AKR1C4 isoform (1.96%). These findings are consistent with previous research showing that the AKR1C3 enzyme can be strongly inhibited by diverse types of compounds isolated from plant sources [52]. We observed inhibitory activity at concentrations as low as 3 µg/mL, indicating the strong potency of the tested extract at low doses. As expected, no change in NADPH fluorescence was observed in the absence of the enzyme (blank), whereas the control reaction showed a decrease in fluorescence due to NADPH consumption, and the presence of a known inhibitor (ibuprofen; IBU) resulted in a reduced slope. The selective inhibition of AKR1C3 by the extract is important, as the inhibition of AKR1C4 is undesirable due to its role in bile acid synthesis, steroid hormone inactivation and elimination [53]. Currently there are no AKR1C3 inhibitors approved for clinical use. However, several potent AKR1C3 inhibitors have been reported, including non-steroidal anti-inflammatory drugs, as well as dietary phytoestrogens, such as quercetin, biochanin and coumestrol [54]. Moreover, in our preliminary studies we observed that steroid derivatives displayed strong AKR1C3 inhibitory activity [44]. The strong and selective inhibition of AKR1C3 by the *A. alpina* extract, combined with its high binding affinity for ERα, suggests the potential dual mechanism of action—decreasing steroid hormone levels and modulating steroid receptor pathways—making it a promising candidate for further investigation in the context of women’s reproductive health and hormone-dependent breast cancer therapy. The inhibition of AKR1C3 by the tested extract also indicates its potential as a natural adjuvant to restore chemosensitivity in drug-resistant breast cancer [55]. However, further studies are necessary to fully assess its therapeutic potential. To better understand which compounds are responsible for the diverse biological activities of *A. alpina* extract observed here, bioactivity-guided fractionation could be a valuable next step.

#### 2.2.8. Assessment of Anti-Inflammatory Activity of Tested *A. alpina* Extract

**Figure 6:**
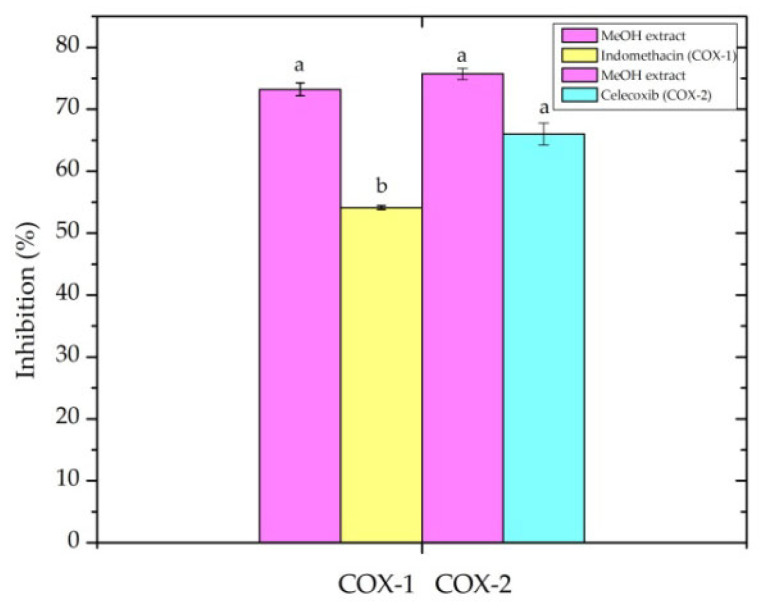
COX-1 and COX-2 inhibitory activity of *Alchemilla alpina* MeOH extract compared with reference compounds. The MeOH extract was tested against both enzymes and compared with indomethacin (COX-1) and celecoxib (COX-2) as standard inhibitors. Data are presented as mean ± standard deviation (SD). Statistical significance was determined by one-way ANOVA followed by Tukey’s post hoc test. Means with different letters (a, b) indicate statistically significant differences (*p* < 0.05).

Acute and chronic inflammations are known to influence both the onset and progression of numerous diseases. As potential treatments, plant extracts that contain various bioactive compounds that reduce inflammatory processes are quite appealing. Continuous attempts have been made to investigate the effectiveness of medicinal plants and their phytochemicals in the treatment and prevention of inflammatory illnesses in order to overcome the drawbacks of synthetic medications, such as non-steroidal anti-inflammatory drugs (NSAIDs). In this situation, the use of herbal remedies is proven to be efficient and less harmful to human body. The present results (Figure 6) revealed a great capacity of *A. alpina* MeOH extract to inhibit the activity of both enzyme isoforms, COX-1 and COX-2.

On the histogram (Figure 6) it can be noticed that the inhibitory potency of the extract against these enzymes is similar (I-COX-1:73.22% and I-COX-2:75.72%). It can also be concluded that the tested extract shows greater inhibitory power compared to the positive control substances, such as indomethacin and celecoxib. However, this comparison is not entirely relevant considering that the concentrations of the extract and these synthetic anti-inflammatory drugs differ. In the previously published results [15], the extracts of the *A. vulgaris* plant show lower inhibitory activity (32.11–62.84%) compared to the results obtained in our study using the same concentration (50 µg/mL).

In the study of Kanak et al. [1], the anti-inflammatory activity of *Alchemilla acutiloba* was investigated. The results showed that all fractions, such as methanolic, butanolic and ethyl acetate (except diethyl ether), of the aerial parts and roots of *A. acutiloba* extract demonstrated great inhibitory effect, especially towards the enzyme COX-2 [1]. Furthermore, Şeker Karatoprak et al. [39] determined the anti-inflammatory activity by measuring the inhibitory effect of *A. mollis* MeOH and water extracts on NO production and pro-inflammatory cytokine TNF-α levels in lipopolysaccharide (LPS)-stimulated mouse RAW macrophages. Both extracts significantly decreased NO in a dose-dependent manner [14].

The pronounced COX inhibitory activity of the *A. alpina* extract indicates the presence of biomolecules that possess anti-inflammatory capacities. Most of the identified compounds belong to the phenolic group, and several of them, including catechin, rutin, kaempherol, caffeic acid and chlorogenic acid, have already been reported in a previous study [55]. Notably, recent evidence shows that isocoumarin derivatives—structurally related to certain phenolic constituents—exert anti-inflammatory effects by inhibiting 5-lipoxygenase and prostaglandin E_2_ synthesis, as well as modulating MAPK and NF-κB signaling pathways, which collectively suppress cytokine-mediated inflammation [56].

Given the absence of evidence regarding the anti-inflammatory properties of this plant’s extracts, our findings present numerous opportunities for subsequent, more comprehensive investigations in this area.

## 3. Materials and Methods

### 3.1. Plant Material and Extract Preparation

For the research, a cultivated dried plant material was used, purchased from Galsters Kräuter (Galsters Kräuter, Schwebheim, Germany), a company specializing in the cultivation and distribution of aromatic, spice, and medicinal plants.

Five grams of the plant material was weighed and mixed with 80 mL of 70% MeOH. Ultrasound-assisted extraction was performed in an ultrasonic bath (Bandelin Sonorex RK 100, Bandelin Electronic GmbH, Berlin, Germany; 35 kHz and 320 W) for 10 min at 20 °C. After sonication, the supernatant was separated and filtered, and the residue was subjected to the same extraction conditions twice more. The combined extracts were evaporated to dryness using a rotary evaporator (Rotavapor R-300, Büchi Labortechnik AG, Flawil, Switzerland), and a stock solution with a concentration of 100 mg/mL was prepared.

### 3.2. Chemical Characterization

#### 3.2.1. Total Phenolic and Total Flavonoid Contents

The total phenolic content (TPC) was determined by using Folin–Ciocalteu (FC) reagent, following the method described in Lesjak et al. [57]. The sample was tested at concentrations of 0.25, 0.5 and 1.0 mg/mL. After 2 h of incubation, the absorbance was measured at 720 nm (Multiscan spectrophotometer, Thermo Scientific, Waltham, MA, USA). The results were expressed as milligrams of gallic acid equivalents per gram of dry weight (mg GAE/g d.w.).

The total flavonoid content (TFC) was determined using the AlCl_3_ complexation method described by Lesjak et al. [57], with the absorbance measured at 415 nm using a multiscan spectrophotometer (Thermo Scientific, Waltham, MA, USA). The samples were tested at concentrations of 0.25, 0.5, and 1.0 mg/mL. The TFC values were expressed as milligrams of quercetin equivalents per gram of dry weight (mg QE/g d.w.).

#### 3.2.2. LC-MS/MS Analysis

The chemical profile of the *A. alpina* MeOH extract was examined using LC–MS/MS. The content of the 43 selected phenolic compounds was determined according to the method described by Orčić et al., with some modifications [58]. The analyses were performed using an Agilent 1200 Series HPLC system connected to a 6410A Triple Quadrupole Mass Spectrometer (Agilent Technologies, Santa Clara, CA, USA), operated with MassHunter Workstation Data Acquisition software (version B.06.00). The extracts were dissolved in 50% MeOH at a concentration of 20 and 2 mg/mL. A 5 μL injection volume was applied for chromatographic separation using a Zorbax Eclipse XDB-C18 rapid resolution HT column (4.6 mm × 50 mm, and 1.8 μm; Agilent Technologies, Santa Clara, CA, USA) thermostated at 50 °C. The mobile phase consisted of the following: A: 0.05% formic acid in water; B: MeOH. Separation was achieved using the following gradient: 0 min 15% B, 2 min 15% B, 19 min 50% B, 24 min 100% B, and 27 min 100% B (post time was 6 min; total time for analysis was 33 min). Data acquisition was carried out in dynamic MRM mode (the parameters are given in Table S1), and quantification was completed using MassHunter Qualitative Analysis software (version B.06.00). Calibration curves were constructed using Origin 2019b (calibration curves equations and R^2^ for all quantified compounds are given in the Supplementary Table S2).

### 3.3. Examination of Biological Activities

#### 3.3.1. *In Vitro* Antioxidant Activity

Antioxidant activity was assessed using four methods: DPPH, ABTS, nitric oxide (NO) radical scavenging, and FRAP assays.

For the DPPH assay, 60 μL of a 90 μM DPPH solution was combined with 180 μL of MeOH and 10 μL of extract (concentration range: 0.046875–3.0 mg/mL) or standard (Trolox: 0.00026–0.0167 mg/mL). After 30 min of incubation in the dark, the absorbance was measured at 515 nm [57].

In the ABTS assay, 290 μL of ABTS reagent (7 mM with 2.45 mM K_2_S_2_O_8_) was mixed with 10 μL of extract (concentration range: 0.046875–3.0 mg/mL), or standard (propyl gallate, PG; concentration range: 0.00010–0.015 mg/mL). After 5 min of incubation, the absorbance was measured at 734 nm [59].

For the NO scavenging assay, 15 μL of extract (concentration range: 0.046875–3.0 mg/mL) or standard (PG; concentration range: 0.010–1.5 mg/mL) was added to 250 μL of sodium nitroprusside solution prepared in phosphate buffer. After 90 min of incubation, 500 μL of Griess reagent was added, and the absorbance was recorded at 546 nm [60].

In the FRAP assay, 10 μL of extract at concentrations of 0.08, 0.15 and 0.3 mg/mL or PG at the same concentrations was mixed with 225 μL of FRAP reagent and 22.5 μL of distilled water (dH_2_O). After 6 min of incubation, the absorbance was measured at 593 nm [57].

The antioxidant capacities determined by the DPPH, ABTS, and NO assays were expressed as IC_50_ values (mg/mL) calculated by linear interpolation from percentage inhibition values obtained using the following formula:

% inhibition = [1 − (A_sample_ − A_correction_)/A_control_] × 100

For the FRAP assay, the results were expressed as milligrams of ascorbic acid equivalents per gram of dry weight (mg AAE/g d.w.). A calibration curve was constructed using ascorbic acid (R^2^ ≥ 0.99), and sample absorbance values were interpolated from this curve and normalized to the dry weight of the extract.

#### 3.3.2. α-Amylase and α-Glucosidase Inhibitory Activity Assays

The *in vitro* enzyme inhibitory potential of the extract was evaluated by determining its inhibitory effects on two digestive enzymes, α-amylase and α-glucosidase, both sourced from Sigma-Aldrich (Steinheim, Germany).

α-Amylase inhibition was assessed following the method of Yang et al. [61]. In brief, 90 μL of α-amylase (porcine pancreas, Type VI–B) was mixed with 80 μL of 0.05% starch solution in 20 mM phosphate buffer (pH 6.9) and 10 μL of extract (0.28 mg/mL) or acarbose (0.1 mg/mL). For the blank, α-amylase was replaced with buffer; for the control, the extract was substituted with buffer. After 10 min of incubation at 37 °C with constant shaking (IKA KS 4000i, IKA^®^-Werke GmbH and Co. KG, Staufen im Breisgau, Baden-Württemberg, Germany), the reaction was stopped with 100 μL of cold 1 M HCl and 20 μL of Lugol’s solution. The absorbance was measured at 620 nm.

For α-glucosidase inhibition, the method of Palanisamy et al. [62] was used. A mixture of 100 μL of 0.1 M phosphate buffer (pH 6.8), 10 μL of α-glucosidase (*Saccharomyces cerevisiae*, Type I), 20 μL of extract (0.003906 mg/mL) or acarbose (0.026 mg/mL), and 20 μL of *p*-nitrophenyl α-D-glucoside was incubated at 37 °C for 15 min with shaking. In the blank, the enzyme was replaced with buffer; in the control, the extract was replaced with buffer. After incubation, 80 μL of 0.2 M Na_2_CO_3_ was added, the absorbance was read at 400 nm, and the results are expressed as % of enzyme inhibition.

#### 3.3.3. Anti-Acetylcholinesterase Activity Assay

The acetylcholinesterase inhibitory activity of the extract was determined using a modified Ellman’s method [63]. In brief, 20 µL of AChE (0.5 U/mL; *Electrophorus electricus* (electric eel), Type VI-S, Sigma-Aldrich (Steinheim, Germany)), 110 µL of 20 mM Tris-HCl buffer (pH 8), and 10 µL of extract (0.5 mg/mL) or eserine standard (0.069 ng/mL) were combined. For the blank, AChE was replaced with Tris-HCl buffer (pH 7.5), while the control contained a buffer (pH 8) instead of the sample. After 15 min of incubation at 37 °C with shaking, 40 µL of 3 mM DTNB and 20 µL of 15 mM acetylthiocholine iodide were added. The absorbance was recorded at 412 nm after 3 min. Tests were performed in triplicate and the results are expressed as % of enzyme inhibition.

#### 3.3.4. Cytotoxicity Evaluation

##### Cell Culture

Human fetal lung fibroblasts (MRC-5 and ATCC), human cervical carcinoma cells (HeLa and ATCC) and human ovarian cancer cells (A2780; a kind gift from Institute for Biological Research “Siniša Stanković”, Belgrade, Serbia) were propagated in 25 cm^2^ tissue culture flasks in a humidified incubator with 5% CO_2_ at 37 °C. MRC-5 and HeLa cells were grown in a complete RPMI medium (RPMI 1640 medium (Biowest, Nuaillé, France), 10% fetal calf serum (FCS, Gibco, Waltham, MA, USA), and 1% antibiotic–antimycotic solution (Capricorn Scientific GmbH, Ebsdorfergrund, Germany), while A2780 was cultured in complete DMEM-F12 medium (Biowest, Nuaillé France). After reaching 70% confluence, the cells were trypsinized (0.25% trypsin–EDTA solution, Biowest, Nuaillé, France), seeded in 96-well plates (1.5 × 10^4^ cells/well) and were allowed to attach to wells for 24 h at 37 °C, 5% CO_2_, before the treatment.

##### Treatment Preparation

The stock solution of the extract was prepared in DMSO at a concentration of 100 mg/mL and which was kept at 4 °C. For the experiment, final concentrations of the extract were prepared from the stock solution by dissolving it in fresh complete cell medium to reach the final concentrations of 6.25, 12.5, 25, 50, and 100 µg/mL. These concentrations were further used for cell treatments.

##### MTT Assay

The MRC-5 and HeLa cells in the complete RPMI medium and A2780 in the DMEM-F12 medium were seeded in 96-well plates at a density of 1.5 × 10^4^ cells/well, with a final volume of 100 µL per well. The medium was exchanged after 24 h, and treatments were added in a total volume of 100 µL/well. Following the incubation with the treatments or solvent (control) at 37 °C for 24 h, an MTT assay was performed. MTT reagent (thiazolyl blue tetrazolium bromide, 1 mg/mL, Sigma Aldrich, St. Louis, MO, USA) was added (10 µL per each well), and the cells were left for 2 h in the dark at 37 °C for the reaction to occur. Furthermore, purple formazan crystals were dissolved with sodium dodecyl sulfate (10% SDS in 0.01 M HCl, Sigma Aldrich, St. Louis, MO, USA). Finally, the absorbance was measured at 570 nm on a microplate reader (Epoch, BioTek, (Agilent Technologies), Winooski, VT, USA) after the complete solubilization of the crystals. The data were expressed as percentage viability in relation to the control (100%). The mean values were represented on bars, from three independent experiments performed in triplicate (n = 9).

#### 3.3.5. Intracellular ROS Production

Cellular oxidative stress due to AAPH-induced ROS was measured by the H_2_DCFDA assay (2′,7′-dichlorofluorescin diacetate). The MRC-5 cells seeded in 96-well plates at a density of 1.5 × 10^4^ cells/well were left overnight to attach to the wells, and kept in a humified incubator at 5% CO_2_ and 37 °C. The next day, the medium was removed and cells were rinsed with PBS. Next, an assay was performed in line with the manufacturer’s instructions. Using PBS as the diluent, 5 μM of the cell-permeable oxidation-sensitive probe H_2_DCFDA (Merck Millipore, Burlington, MA, USA; 2′,7′-dichlorofluorescin diacetate, CAS 4091-99-0; Calbiochem) was added to the cells and left for 45 min in the dark. Next, the cells were washed with PBS and exposed to 25 mM 2,2′-azobis(2-amidinopropane) dihydrochloride (AAPH) alone (to induce ROS overproduction in the cells) or to the combination of AAPH and the extract at a range of concentrations (6.25, 12.5, 25, 50, and 100 µg/mL), and another set of cells was exposed to the extract alone at the same range of concentrations. After an incubation time of 1 h, and the conversion of non-fluorescent H_2_DCFDA to the highly fluorescent 2′, 7′-dichlorofluorescein (DCF), the generation of the intracellular ROS level in cells was determined by measuring the fluorescence on a fluorescent plate reader (Wallac 1420 multilabel counter Victor 3V, Turku, Finland) at excitation and emission wavelengths of 485 and 535, respectively. The data were expressed as the relative fluorescence intensity representing the measured signal intensity detected by the plate reader, and mean value was represented on figures, from three independent experiments performed in triplicate (n = 9).

#### 3.3.6. Evaluation of Receptor Binding and AKR1C Enzyme Inhibition

##### Fluorescent Screen in Yeast

Following previously published procedures for the fluorescent assay in yeast [25,42,43], the relative binding affinities of *A. alpina* MeOH extract for ERα-, ERβ-, AR- and GR-LBD were evaluated. ERα-, ERβ-, AR- and GR-LBD were fused to YFP and expressed in *Saccharomyces cerevisiae*. The FY250 yeast strain (MATα, ura3-52, his3Δ200, leu2Δ1, and trp1Δ63) and the plasmid constructs pRF4–6-ERα LBD-EYFP, pRF4–6-ERβ LBD-EYFP, pRF4–6-AR LBD-EYFP and pRF4–6-GR LBD-EYFP were generously provided by Dr. Blake Peterson from the University of Kansas. Yeast transformation was performed via the standard lithium acetate method [64]. Transformed yeast cells were grown to saturation in selection media without tryptophan (6.7 g/L yeast nitrogen base w/o amino acids and ammonium sulfate; 1.92 g/L yeast synthetic drop-out medium supplement), supplemented with 2% raffinose at 28 °C with shaking (BioSan orbital shaker–incubator ES-20/60, BioSan SIA, Riga, Latvia). Following dilution, the cultures were grown to mid-log phase OD_600nm_~0.4–0.6, and protein expression was induced by addition of 2% galactose. Simultaneously, with galactose induction, the extract freshly dissolved in DMSO was added to the cultures and tested at three concentrations: 0.1, 0.5, and 1 mg/mL. To ensure the assay’s reliability, both positive and negative controls were included for each receptor type. Specifically, estrone/androstenedione (ERα and ERβ), androstenedione/estrone (AR), and prednisolone/estradiol (GR) were used as positive/negative controls, respectively. Control ligands were used at 10 µM in the ERα/ERβ assays, and at 100 µM in the AR/GR assays. Additional controls included non-induced cells (grown in the absence of galactose) and cells treated with DMSO only. Incubation was continued in the dark for 14–16 h at 23 °C. Prior to detection, yeast cultures were transferred to a 96-well microplate. Fluorescence was measured using a Fluoroskan Ascent FL fluorometer (Thermo Fisher Scientific, Waltham, MA, USA), with the excitation and emission wavelengths set at 485 and 538 nm, respectively. The measurement of OD_600nm_ was performed on ThermoLabsystems Multiscan EX spectrophotometer (Thermo Fisher Scientific, Waltham, MA, USA). All the experiments were conducted in triplicate. The relative binding affinities were calculated as the fold fluorescence change between ligand-treated cells and the control cells treated with DMSO (ER and AR assays) or estradiol (GR assay), normalized to one. Histograms were generated using Origin Pro 8.0 (OriginLab Corporation, Northampton, MA, USA). Error bars correspond to the propagated standard errors of the mean.

##### Expression and Purification of Human Recombinant AKR1C3 and AKR1C4

The genes encoding human AKR1C3 and AKR1C4 were cloned into pET28b(+) plasmids (a kind gift from Dr. Chris Bunce, University of Birmingham), which were used to transform *Escherichia coli* BL21 (DE3) cells for recombinant protein expression, as previously described [25,44,45]. Chemically competent cells were transformed using the heat shock method. Cultures were grown overnight at 37 °C in LB medium supplemented with kanamycin (50 µg/mL) until saturation; then, they were diluted into fresh medium and incubated until they reached OD_600nm_~0.4–0.6 (BioSan orbital shaker–incubator ES-20/60 (BioSan SIA, Riga, Latvia). At this point, protein expression was induced by the addition of 0.5 mM IPTG. After induction, the cultures were incubated for 20 h at 23 °C. The cells were then harvested via centrifugation (5000× *g*, 10 min, and 4 °C) using a Centrifuge 5810 R (Eppendorf, Hamburg, Germany), resuspended in lysis buffer (20 mM Tris-HCl pH 8.0, 5 mM imidazole, and 1 mg/mL lysozyme) and lysed by three freeze–thaw cycles followed by seven cycles of sonication on ice using a Soniprep 150 ultrasonic homogenizer (MSE, London, UK; 30 s pulses). All purification steps were performed at 4 °C. After centrifugation (12,000× *g*, 45 min, and 4 °C) of the lysate, the supernatant containing soluble AKR1C3 or AKR1C4 was loaded onto a 1 mL HisTrap HP column (GE Healthcare, Chicago, IL, USA) coated with Ni sepharose. The column was equilibrated with a binding buffer (20 mM Tris-HCl, pH 8.0, and 0.5 M NaCl) and bound His-tagged protein was eluted with 400 mM imidazole. The eluted protein was further purified by size-exclusion chromatography using a Bio-Gel P-10 column (Bio-Rad, Hercules, CA, USA) to remove any contaminants and imidazole. At the final step of purification, fractions containing the highest protein concentration were pooled and stored at −80 °C until use.

##### AKR1C3 and AKR1C4 Inhibition Assay

The inhibition potential of *A. alpina* MeOH extract against human recombinant AKR1C3 and AKR1C4 was evaluated using an NADPH consumption assay by monitoring the decrease in NADPH fluorescence, as previously described [25,44,45]. The reactions were carried out in 300 µL reaction volumes consisting of 80 μg/mL AKR1C3 or 160 μg/mL AKR1C4, 9,10-phenanthrenequinone (4 µM for AKR1C3 and 17 µM for AKR1C4) and 250 μM NADPH in 100 mM potassium phosphate buffer (pH 6.0). The inhibitory effect of the extract was tested at a concentration of 3 µg/mL. Ibuprofen (33 µM), a well-known AKR1C inhibitor, was used as a reference. The enzyme was preincubated with ibuprofen or the extract for 15 min at 37 °C. The reactions were conducted in triplicate and initiated upon the addition of substrate and NADPH. The enzyme assay was conducted at 37 °C, with the fluorescence measurements recorded every 30 s over a 10 min period. Substrate utilization was monitored via the decrease in the fluorescence of NADPH using a Fluoroskan Ascent FL florometer (ex 340 nm/em 460 nm, Thermo Fisher Scientific, Waltham, MA, USA). Raw fluorescence values were first normalized to the initial fluorescence value for each sample. The normalized fluorescence data obtained from triplicate measurements were averaged and fitted by linear regression over the measurement interval. The resulting regression equations were used to describe the time-dependent decrease in NADPH fluorescence, yielding fluorescence curves effectively starting from a normalized value of one. The inhibition potential was calculated from the slope of the fluorescence–time curve, with the enzyme activity in the absence of inhibitor defined as 100%. The percent inhibition of AKR1C3/AKR1C4 activity was calculated using the following equation: % inhibition = 100% − [slope(test)/slope (reaction) × 100], where slope(test) represents the slope obtained in the presence of ibuprofen or extract, and slope (reaction) is the control (the reaction without the inhibitor). The blank was prepared by omitting the enzyme from the reaction mixture.

#### 3.3.7. COX Anti-Inflammatory Activity

Anti-inflammatory activity was evaluated using a commercial kit (Enzo Life Sciences, Farmingdale, NY, USA) based on the quantitative determination of prostaglandin E_2_ (PGE_2_), formed in an *in vitro* system from arachidonic acid (1 mM) with the addition of COX-1 (ovine; Cayman Chemical, Ann Arbor, MI USA, 60100) and COX-2 (human recombinant; Cayman Chemical, Ann Arbor, MI, USA, 60122) enzymes (0.2 U/well), as published by Katanić Stanković et al. [65]. The experiment was performed in 96-well microplates. Along with COX-1/2, arachidonic acid, and the tested MeOH extract, the following reagents were included in the reaction mixture: EDTA-Na_2_ (Titriplex^®^ III, Merck KGaA, Darmstadt, Germany; 1 mM) dissolved in 0.1 M TRIS/HCl buffer, adrenaline bitartrate (18 mM), and hematin (100 µM). The reaction was terminated by the addition of 10% (*v*/*v*) formic acid. Indomethacin (25 μM) and celecoxib (176 μM) served as the selective inhibitors for COX-1 and COX-2, respectively. The quantitative determination of PGE_2_ was carried out according to the manufacturer’s instructions for the Enzo PGE_2_ ELISA kit. After incubation and the termination of the reaction, the absorbance was read at 405 nm using a Hidex microplate reader (Turku, Finland). The results were expressed as the percentage inhibition of the COX-1/COX-2 enzyme. Non-steroidal anti-inflammatory drugs (NSAID), indomethacin/celecoxib, were used as the positive controls.

### 3.4. Statistical Analysis

All the assays were conducted in triplicate, and the results were presented as the mean ± standard deviation (SD). Data with a normal distribution were subjected to both univariate (ANOVA) and multivariate (MANOVA) analyses. The Tukey Honestly Significant Difference (HSD) test was applied to determine the statistically significant differences among the analyzed extracts at a 95% confidence level (*p* < 0.05). GraphPad Prism 6.0 (GraphPad Software, Inc., La Jolla, CA, USA) was used for the statistical analysis of cell-based assays, where *p* < 0.05 was considered significant. Figure 1 and Figure 2 were prepared using GraphPad Prism 6.0 (GraphPad Software, San Diego, CA, USA), and Figure 3, Figure 4, Figure 5 and Figure 6 were prepared using OriginPro 8.0 (OriginLab Corporation, Northampton, MA, USA).

## 4. Conclusions

The obtained results suggest the strong antioxidant, anti-inflammatory, hypoglycemic, and estrogen receptor-modulating properties of *Alchemilla alpina* MeOH extract. The extract exhibited a concentration-dependent reduction in cell viability in both non-tumorigenic MRC-5 and the cancer cells HeLa and A2780, with no clear selectivity toward malignant phenotypes. Additionally, the extract demonstrated significant antioxidant activity by lowering intracellular ROS levels in AAPH-exposed MRC-5 cells, indicating cytoprotective effects under oxidative stress conditions. Such pronounced biological activity of the investigated *A. alpina* extract corresponds well with its high phenolic content, particularly rutin, catechin, caffeic acid, and chlorogenic acid.

Furthermore, the MeOH extract showed the strong and selective inhibition of human recombinant AKR1C3, along with high binding affinity for ERα, comparable to the natural ligand estrone. These findings suggest a dual mechanism of action, emphasizing *A. alpina* as a promising natural source for supporting women’s reproductive health and as a potential candidate for developing agents targeting the modulation of estrogen signaling. However, its potential in hormone-dependent reproductive cancers is yet to be explored. Overall, *A. alpina* represents a valuable and prospective source of therapeutically relevant bioactive compounds, providing biological and phytopharmacological support for the traditional use of *Alchemilla* spp. to treat reproductive and metabolic disorders. Nevertheless, further studies involving bioassay-guided fractionation, mechanistic exploration, and *in vivo* validation are essential to identify the active constituents and elucidate their precise molecular targets, thereby confirming and expanding the observed pharmacological potential.

## Figures and Tables

**Table 1:** Total phenolic and total flavonoid contents of *A. alpina* extract and antioxidant activity of the extract and reference standards (Trolox and propyl gallate).

Test	MeOH Extract of *A. alpina*	Standard Antioxidant *
Total phenolic (mg GAE/g d.w.)	163.12 ± 1.62	na
Total flavonoid content (mg QE/g d.w.)	40.48 ± 0.49	na
DPPH (IC_50_ (mg/mL))	0.01188 ± 0.001 ^b^	0.00011 ± 0.00001 ^a^
ABTS (IC_50_ (mg/mL))	0.032 ± 0.001 ^b^	0.0012 ± 0.0001 ^a^
FRAP (mg AAE/g d.w.)	158.46 ± 13.25 ^b^	215.01 ± 4.43 ^a^
NO (IC_50_ (mg/mL))	1.15 ± 0.24 ^a^	0.81 ± 0.12 ^a^

Data are presented as the mean ± standard deviation (SD). Significant differences were determined by a one-way ANOVA followed by Tukey’s post hoc test. Means with different superscript letters (a, b) indicate statistically significant differences (*p* ˂ 0.05). * Trolox was used as the standard antioxidant in the DPPH assay, whereas propyl gallate was employed as the reference antioxidant in the ABTS, FRAP, and NO assays. Abbreviations: d.w.—dry weight; GAE—gallic acid equivalents; QE—quercetin equivalents; AAE—ascorbic acid equivalents; DPPH—2,2-diphenyl-1-picrylhydrazyl; ABTS—2,2′-azino-bis (3-ethylbenzothiazoline-6-sulfonic acid); FRAP—ferric reducing antioxidant power; NO—nitric oxide; and na—not applicable.

**Table 2:** LC–MS/MS analysis of selected phenolic compounds and quinic acid in MeOH extracts of *Alchemilla alpina* (µg/g dry weight, d.w.).

Analyzed Compounds	Content (µg/g d.w.)
*Hydroxybenzoic acid derivatives*	
*p*-Hydroxybenzoic acid	67.12 ± 0.76
Protocatechuic acid	54.93 ± 0.46
Gentisic acid	22.54 ± 0.27
Vanillic acid	26.61 ± 0.37
Gallic acid	96.99 ± 7.14
Syringic acid	5.43 ± 0.15
*Hydroxycinnamic acid derivatives*	
Cinnamic acid	11.91 ± 0.07
3,4-dimethoxycinnamic acid	n.d.
*p*-Coumaric acid	55.60 ± 1.14
*o*-Coumaric acid	0.40 ± 0.03
Caffeic acid	181.28 ± 4.37
Ferulic acid	58.09 ± 0.98
Sinapic acid	4.03 ± 0.14
Chlorogenic acid	163.99 ± 53.81
*Flavonols*	
Quercetin	45.23 ± 0.70
Quercetin-3-*O*-galactoside	64.70 ± 1.72
Quercetin-3-*O*-glucoside	127.96 ± 0.62
Quercitrin	19.16 ± 5.99
Kaempferol	26.95 ± 1.68
Kaempferol-3-*O*-glucoside	318.30 ± 11.26
Myricetin	n.d.
Isorhamnetin	14.81 ± 1.03
Rutin	527.83 ± 8.31
*Flavones*	
Apigenin	3.13 ± 0.03
Apigenin-7-*O*-glucoside	1.21 ± 0.08
Luteolin	44.79 ± 1.23
Luteolin-7-*O*-glucoside	123.84 ± 3.02
Vitexin	3.42 ± 0.04
Chrysoeriol	6.78 ± 0.10
Baicalein	<1.2
Baicalin	n.d.
Amentoflavone	<9.75
Apiin	<1.2
*Flavan-3-ols (catechins)*	
Catechin	347.16 ± 12.15
Epicatechin	3.49 ± 0.26
Epigallocatechin gallate	<4.9
*Flavanones*	
Naringenin	2.18 ± 0.02
*Isoflavones*	
Genistein	1.89 ± 0.09
Daidzein	n.d.
*Coumarins*	
Esculetin	10.85 ± 0.18
Umbelliferone	0.44 ± 0.04
Scopoletin	0.45 ± 0.06
*Lignans*	
Matairesinol	n.d.
Secoisolariciresinol	˂0.6

Data are presented as the mean ± standard deviation (SD). Values denoted with the symbol “<” indicate concentrations below the limit of quantification (LoQ) but above the limit of detection (LoD), meaning the compound was detected but not reliably quantified. Abbreviation: n.d.—not detected.

**Table 3:** The enzyme inhibition activities of the tested *A. alpina* MeOH extract and reference standard compounds (acarbose and eserine), expressed as percentage (%) of inhibition.

Enzyme Inhibition	Tested MeOH Extract	Tested Standard Compound
		Acarbose
α-amylase (%)	8.30 ± 0.46 ^b^	72.41 ± 6.12 ^a^
α-glucosidase (%)	48.34 ± 2.90 ^a^	28.06 ± 2.66 ^b^
		Eserine
Anti-acetylcholinesterase (%)	94.94 ± 0.65 ^a^	95.51 ± 1.12 ^a^

Data are represented as the mean ± standard deviation (SD). Significant differences were determined by a one-way ANOVA followed by Tukey’s post hoc test. Means with different superscript letters (a, b) indicate statistically significant differences (*p* < 0.05). The activity of acarbose was determined at concentration of 0.1 mg/mL for α-amylase and 0.026 mg/mL for α-glucosidase, whereas the activities of the extracts were tested at 0.28 mg/mL and 0.003906 mg/mL for α-amylase and α-glucosidase, respectively. The anti-acetylcholinesterase activity of eserine was tested at a concentration of 0.069 ng/mL, while the extract was tested at concentration of 0.5 mg/mL.

## Data Availability

Data are contained within the article and Supplementary Materials.

## Supplementary Materials

The following supporting information can be downloaded at https://www.mdpi.com/article/10.3390/ijms27073025/s1.
