# Supplementary material for: Bioactivities of Alchemilla alpina L. Extract on Women’s Reproductive and Metabolic Health: Antioxidant, Enzyme Inhibitory, Receptor Modulatory Properties and Potential Cytotoxic Effects
Source: Int J Mol Sci. 2026 Mar 26;27(7):3025. doi: 10.3390/ijms27073025 (PMC13072724; doi:10.3390/ijms27073025)
Supplement: Supplementary file 1 [file ijms-27-03025-s001.zip › ijms-4141380-supplementary.pdf]

International Journal of Molecular Sciences

MDPI

Supplementary material

Table S1. MRM parameters of analysed compounds and LOD and LOQ

Compound	tR [min]	Precursor [m/z]	Fragment [m/z]	Vfragmentor [V]	Vcollision [V]	LOD [ng/mL]	LOQ [ng/mL]
Hydroxybenzoic acid derivatives							
p-Hydroxybenzoic acid	6.6	137	93	80	10	3.05	12.21
Protocatechuic acid	4.1	153	109	105	9	1.53	1.53
Gentisic acid	5.9	153	109	100	9	1.53	1.53
Vanillic acid	8.9	167	108	100	15	24.41	24.41
Gallic acid	2.1	169	125	90	10	1.53	1.53
Syringic acid	10.2	197	182	90	7	6.10	6.10
Hydroxycinnamic acid derivatives							
Cinnamic acid	20.2	147	103	100	5	48.83	48.83
3,4-dimethoxycinnamic acid	17.7	207	103	110	7	12.21	12.21
p-Coumaric acid	11.5	163	119	90	9	1.53	1.53
o-Coumaric acid	15.9	163	119	100	5	1.53	1.53
Caffeic acid	8.7	179	135	100	10	1.53	1.53
Ferulic acid	13.2	193	134	90	11	1.53	1.53
Sinapic acid	13.8	223	193	100	17	3.05	3.05
Chlorogenic acid	8.1	353	191	100	10	1.53	1.53
Flavonols							
Quercetin	20.4	301	151	130	15	24.41	195.31
Quercetin-3-*O*-galactoside	15.5	463	300	200	30	1.53	3.05
Quercetin-3-*O*-glucoside	15.9	463	300	210	30	1.53	3.05
Quercitrin	17.6	447	300	190	27	1.53	24.41
Kaempferol	22.2	285	285	130	0	3.05	48.83
Kaempferol-3-*O*-glucoside	17.7	447	284	190	30	1.53	6.10
Myricetin	17.2	317	179	150	20	97.66	195.31
Isorhamnetin	22.4	315	300	160	21	3.05	97.66
Rutin	15.9	609	300	135	42	1.53	1.53
Flavones							
Apigenin	22.3	269	117	130	25	1.53	24.41
Apigenin-7-*O*-glucoside	17.3	431	268	135	41	1.53	12.21
Luteolin	21.2	285	133	135	25	1.53	48.83
Luteolin-7-*O*-glucoside	15.6	447	285	230	30	1.53	12.21
Vitexin	14.8	431	311	200	22	1.53	1.53
Chrysoeriol	22.5	299	284	125	20	1.53	24.41
Baicalein	22.8	269	269	165	0	1.53	196.31
Baicalin	19.2	445	269	140	22	1.53	24.41
Amentoflavone	23.2	537	375	220	35	1.53	195.31
Apiin	17.3	563	269	250	36	1.53	12.21
Flavan-3-ols							
Catechin	7.0	289	245	150	10	12.21	12.21
Epicatechin	10.1	289	245	150	10	6.10	6.10
Epigallocatechin gallate	9.1	457	169	165	16	24.41	97.66
Flavanones							

Naringenin	20.8	271	151	130	16	1.53	1.53
Isoflavones							
Genistein	21.4	269	133	145	32	1.53	1.53
Coumarins							
Esculetin	8.5	177	133	105	15	1.53	1.53
Umbelliferone	12.0	161	133	120	19	3.05	3.05
Scopoletin	12.7	191	176	80	8	1.53	1.53
Lignans							
Matairesinol	20.3	357	122	130	24	6.10	6.10
Secoisolariciresinol	17.9	361	165	130	26	3.05	12.21

Table S2. Calibration curves and R^2 for quantified compounds

Compound	Calibration curve equation	R^2
Hydroxybenzoic acid derivatives		
p-Hydroxybenzoic acid	y = 13.61163x + 437.08696	0.99985
Protocatechuic acid	y = 23.59741x + 143.73913	0.99881
Gentisic acid	y = 16.60394x – 396.08696	0.99724
Vanillic acid	y = 1.42171x – 25.04348	0.99975
Gallic acid	y = 3573 + 18.37502*x -3.36955E-4*x^2	0.99464
Syringic acid	y = 5.35953x - 39.6087	0.99792
Hydroxycinnamic acid derivatives		
Cinnamic acid	y = 1.02329x - 25.73913	0.99849
p-Coumaric acid	y = 47.00211x + 302	0.99979
o-Coumaric acid	y = 35.88238x - 7.3913	0.99991
Caffeic acid	y = 40.33969x + 25779.13043	0.99558
Ferulic acid	y = 15.40857x - 347.65217	0.99938
Sinapic acid	y = 6.20633x - 42.95652	0.99124
Chlorogenic acid	y = 56.11078x - 618.30435	0.99966
Flavonols		
Quercetin	y = 22.48392x - 4012.43478	0.99703
Quercetin-3-*O*-galactoside	y = 36.83996x + 1052.21739	0.99907
Quercetin-3-*O*-glucoside	y = 35.11748x + 3932.26087	0.99835
Quercitrin	y = 43.32384x + 533.08696	0.99839
Kaempferol	y = 10442.5 + 58.46967*x + 0.02141*x^2	0.9996
Kaempferol-3-*O*-glucoside	y = 23.77958x + 9685	0.99746
Isorhamnetin	y = 39.97634x + 958.95652	0.99719
Rutin	y = 37.8003x + 3821.34783	0.99757
Flavones		
Apigenin	y = 39.01066x - 31.78261	0.99824
Apigenin-7-*O*-glucoside	y = -476.5 + 90.83541*x - 0.20744*x^2	0.99779
Luteolin	y = 33.04697x + 1409.34783	0.99682
Luteolin-7-*O*-glucoside	y = 79.28711x - 4305.69565	0.99999
Vitexin	y = 56.6548x - 125.65217	0.99998
Chrysoeriol	y = 61.70695x + 344.86957	0.99844
Flavan-3-ols		
Catechin	y = 2.41029x + 292.95652	0.99878

Epicatechin	y = 4.67407x - 45.17391	0.99417
Flavanones		
Naringenin	y = 20.6125x - 18.56522	0.99795
Isoflavones		
Genistein	y = 11.95462x + 4.26087	0.99928
Coumarins		
Esculetin	y = 31.25159x - 21.08696	0.99756
Umbelliferone	y = 19.6836x - 6.26087	0.99542
Scopoletin	y = 30.75918x + 3.73913	0.99839
